# Large‐scale patterns of seed removal by small mammals differ between areas of low‐ versus high‐wolf occupancy

**DOI:** 10.1002/ece3.6415

**Published:** 2020-06-01

**Authors:** Jennifer L. Chandler, Timothy R. Van Deelen, Nathan P. Nibbelink, John L. Orrock

**Affiliations:** ^1^ Department of Integrative Biology University of Wisconsin Madison Wisconsin USA; ^2^ Department of Forest and Wildlife Ecology University of Wisconsin Madison Wisconsin USA; ^3^ Warnell School of Forestry and Natural Resources University of Georgia Athens Georgia USA

**Keywords:** granivory, predation, small mammal, spatial heterogeneity, top‐down effects

## Abstract

Because most tree species recruit from seeds, seed predation by small‐mammal granivores may be important for determining plant distribution and regeneration in forests. Despite the importance of seed predation, large‐scale patterns of small‐mammal granivory are often highly variable and thus difficult to predict. We hypothesize distributions of apex predators can create large‐scale variation in the distribution and abundance of mesopredators that consume small mammals, creating predictable areas of high and low granivory. For example, because gray wolf (*Canis lupus*) territories are characterized by relatively less use by coyotes (*C. latrans*) and greater use by foxes (*Vulpes vulpes*, *Urocyon cinereoargentus*) that consume a greater proportion of small mammals, wolf territories may be areas of reduced small‐mammal granivory. Using large‐scale, multiyear field trials at 22 sites with high‐ and low‐wolf occupancy in northern Wisconsin, we evaluated whether removal of seeds of four tree species was lower in wolf territories. Consistent with the hypothesized consequences of wolf occupancy, seed removal of three species was more than 25% lower in high‐wolf‐occupancy areas across 2 years and small‐mammal abundance was more than 40% lower in high‐wolf areas during one of two study years. These significant results, in conjunction with evidence of seed consumption in situ and the absence of significant habitat differences between high‐ and low‐wolf areas, suggest that top‐down effects of wolves on small‐mammal granivory and seed survival may occur. Understanding how interactions among carnivores create spatial patterns in interactions among lower trophic levels may allow for more accurate predictions of large‐scale patterns in seed survival and forest composition.

## Graphical abstract

We hypothesize distributions of apex predators can create large‐scale variation in the distribution and abundance of mesopredators that consume small mammals, creating predictable areas of high and low granivory. For example, because gray wolf (*Canis lupus*) territories are characterized by relatively less use by coyotes (*C. latrans*) and greater use by foxes (*Vulpes vulpes*, *Urocyon cinereoargentus*) that consume a greater proportion of small mammals, wolf territories may be areas of reduced small‐mammal granivory. Using multi‐year, large‐scale seed‐removal assays and small‐mammal surveys across landscapes within and between 11 wolf territories in northern Wisconsin, we demonstrated seed removal of three seed species was lower in high‐wolf‐occupancy areas across 2 years and small‐mammal abundance was lower in high‐wolf areas during one of the two study years, suggesting the occurrence of top‐down effects of wolves on small‐mammal granivory and seed survival.

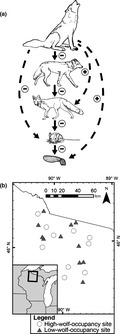

## 1 INTRODUCTION

The distribution and abundance of plants depends upon the survival of seeds (Clark, Poulsen, Levey, & Osenberg, 2007; Crawley, 2000; Orrock, Levey, Danielson, & Damschen, 2006; Turnbull, Crawley, & Rees, 2000). Given the potential importance of seed predation in shaping plant populations and communities, it is important to understand whether there are general, predictable trends in seed predation (as with other important biotic interactions; Schemske, Mittelbach, Cornell, Sobel, & Roy, 2009). Recent evidence suggests that large‐scale (e.g., continental) variation in productivity, seasonality, or latitude may be important for predicting patterns of seed predation (Kelt, Meserve, & Gutiérrez, 2004; Moles, Bonser, Poore, Wallis, & Foley, 2011; Moles & Westoby, 2003; Orrock et al., 2015). While these studies demonstrate the potential for biogeographic patterns in seed removal, they also demonstrate that considerable unexplained variation in seed predation exists at both local and regional scales (i.e., sites within the same biogeographic area) where there are no clear gradients in productivity or seasonality. One possible explanation for this regional‐scale variation is that interactions among apex predators may generate spatial variation in the abundance and activity of granivores, leading to predictable changes in seed predation that would otherwise appear idiosyncratic.

While predators can regulate small‐mammal populations through consumption (Erlinge et al., 1983), predator presence can also affect small‐mammal behavior (e.g., increased vigilance, changes in habitat use) leading to changes in granivory (Brinkerhoff, Haddad, & Orrock, 2005; Brown, 1988; Kotler, Brown, & Hasson, 1991). Small mammals may exhibit antipredator behaviors (e.g., reduced foraging activity and increased use of dense vegetative cover as refuge while foraging; Brinkerhoff et al., 2005; Brown, 1988; Kotler et al., 1991) in response to indications of elevated risk of predation such as predator odors (Apfelbach, Blanchard, Blanchard, Hayes, & McGregor, 2005; Kats & Dill, 1998). Granivorous small mammals can be important agents of seed mortality in a variety of ecosystems (Bricker & Maron, 2012; Clark et al., 2007; Crawley, 2000; Maron, Pearson, Potter, & Ortega, 2012; Orrock et al., 2006), including northern temperate forests (Hulme, 1998), and can alter tree recruitment, abundance, and diversity by reducing survival of tree seeds (Ostfeld, Manson, & Canham, 1997; Whelan, Willson, Tuma, & Souza‐Pinto, 1991). Therefore, predicting forest composition and regeneration may depend on understanding patterns of risk to small mammals at the scale of the distribution and activity of key predators of small mammals in addition to many other known factors that influence seed predation by small mammals (e.g., resource availability, interspecific interactions among small‐mammal species, and plant community composition; Lobo, 2014; Manson, Ostfeld, & Canham, 1998; Schnurr, Canham, Ostfeld, & Inouye, 2004; Schnurr, Ostfeld, & Canham, 2002). However, studies of granivory often are conducted during a single year or at a limited geographical extent, restricting quantification of spatial patterns in seed survival over annual variation since small‐mammal populations are known to fluctuate with weather (Dhawan, Fischhoff, & Ostfeld, 2018; Wang et al., 2009) and resource availability (e.g., seed rain; Ostfeld, Jones, & Wolff, 1996; Pucek, Jędrzejewski, Jędrzejewska, & Pucek, 1993). Despite significant annual variation due to resource availability, weather, or interspecific competition, we propose that multiyear, regional‐scale patterns of granivory may be predictable based upon patterns in the distribution of apex predators.

**FIGURE 1:**
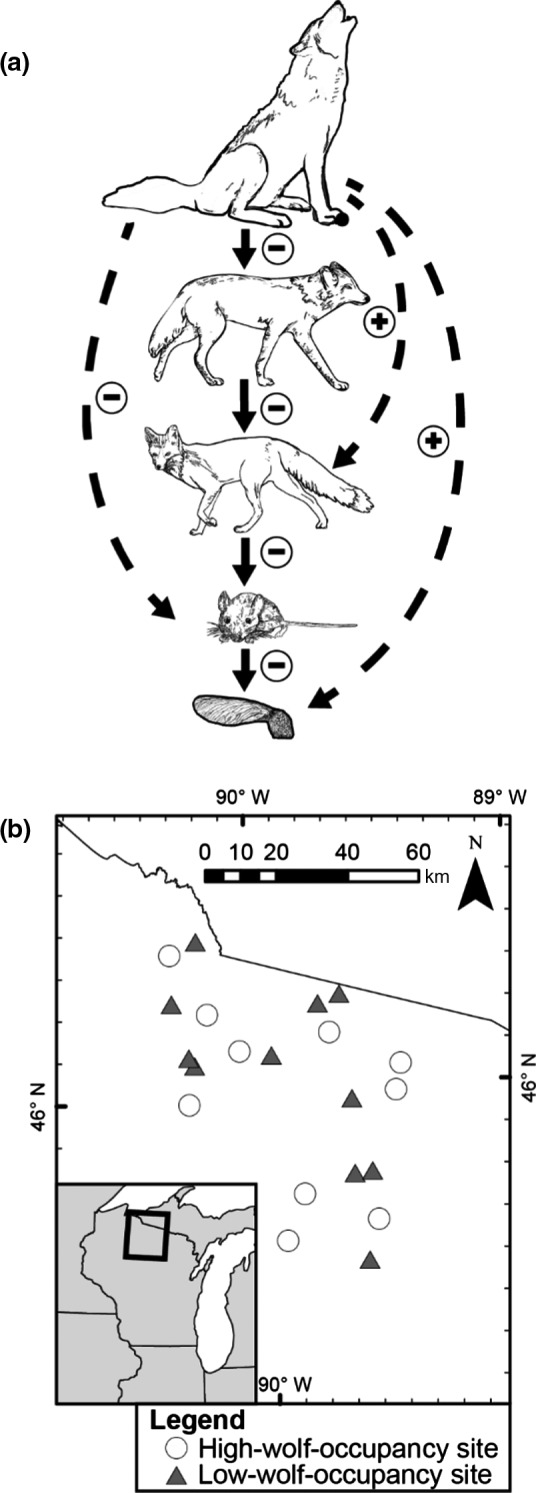
(a) Diagram of how patterns of seed removal and predation might differ in areas with high versus low use by gray wolves (*Canis lupus*), as wolves may change the activity and abundance of small‐mammal predators such as coyotes (*C. latrans*) and foxes (*Vulpes vulpes* and *Urocyon cinereoargenteus*; Flagel et al., 2017). Solid arrows represent direct interactions; broken arrows represent indirect interactions. (b) Study sites in northern Wisconsin, USA. White circles represent sites with high‐wolf occupancy in 2014, and gray triangles represent sites with low‐wolf occupancy in 2014

For example, wolves (*Canis lupus*) competitively exclude coyotes (*C. latrans*; Berger & Gese, 2007; Merkle, Stahler, & Smith, 2009; Switalski, 2003) but not foxes (*Vulpes vulpes*, *Urocyon cinereoargentus*), thereby releasing foxes from competition and predation by coyotes (Flagel, Belovsky, Cramer, Beyer, & Robertson, 2017; Levi & Wilmers, 2012; Major & Sherburne, 1987; Newsome & Ripple, 2015, but see Crimmins & Van Deelen, 2019). Foxes consume greater proportions of small mammals than coyotes (Major & Sherburne, 1987; Tremblay, Crête, & Huot, 1998). Therefore, we expect release of foxes from competition with coyotes to result in a substantial increase in predation of small mammals and to decrease seed survival (Figure 1a). Importantly, areas with high‐wolf activity have lower coyote and higher fox activity, and may predict areas of low deer mouse (*Peromyscus* spp.) abundance (Flagel et al., 2017). Although seed predation by small mammals can shape the survival and distribution of woody plants (Ostfeld et al., 1997; Whelan et al., 1991) and interactions among wolves, coyotes, and foxes have been extensively documented (Flagel et al., 2017; Levi & Wilmers, 2012; Major & Sherburne, 1987; Newsome & Ripple, 2015; Switalski, 2003), it is not known whether these interactions lead to predictable patterns of seed predation.

In this study, we combine multiyear, large‐scale seed‐removal assays and small‐mammal surveys across landscapes within and between 11 wolf territories to evaluate whether small‐mammal abundance, activity, and seed removal differ between areas with high‐ and low‐wolf occupancy. We also compare seed removal between sheltered and unsheltered microhabitats to assess whether risk perceived by small mammals is influenced by wolf occupancy (Brown, 1988; Orrock, Danielson, & Brinkerhoff, 2004). We hypothesize that (a) small‐mammal abundance and seed removal are negatively affected by wolf occupancy across multiple years and (b) seed removal is lowest in unsheltered microhabitats in wolf‐occupied areas.

## 2 MATERIAL AND METHODS

Our study was distributed across approximately 5,000 km^2^ in northern Wisconsin, USA (Figure 1b). Within the study area, we selected 22 50 × 50‐m plots of publicly owned forest dominated by sugar maple (*Acer saccharum*), red maple (*A. rubrum*), balsam fir (*Abies balsamea*), and big‐tooth aspen (*Populus grandidentata*), in high‐ and low‐wolf areas (Figure 1b). We expected higher coyote densities and lower fox densities outside of wolf territories compared to within wolf territories based upon previous studies (Crabtree & Sheldon, 1999; Flagel et al., 2017; Levi & Wilmers, 2012; Newsome & Ripple, 2015), including one study conducted within our study system, within 20 km of our study area (Flagel et al., 2017). We calculated a duration‐weighted wolf‐occupancy index for each site, which gives greater weight to more recent wolf occupancy and has successfully captured wolf‐mediated trophic cascades in a previous study (Callan, 2010; Appendix S1, Equation A1). We chose sites equally distributed between locations with high‐ and low‐duration‐weighted wolf occupancy. High‐wolf‐occupancy sites had a significantly higher wolf‐occupancy index than low‐wolf occupancy sites in both 2014 (*t*_19_ = 18.92, *p* < .01) and 2015 (*t*_19_ = 14.89, *p* < .01; wolf occupancy classification changed for two sites between the 2 years of study). Sites were also stratified across a latitudinal gradient to account for factors that vary with latitude and could affect large‐scale variation in seed removal (Orrock et al., 2015; Figure 1b).

To assess habitat characteristics that might influence small‐mammal activity and seed predation across high‐ and low‐wolf sites, we surveyed understory and shrub‐layer vegetation, basal area (BA) of canopy trees, presence of coarse woody debris near seed depots, canopy cover (using a densiometer), and percent canopy light transmittance (Bolstad & Gower, 1990) at 1.5 and 0.1 m from the forest floor. Each habitat characteristic was assessed once during the 2‐year study. We also evaluated the proportion of the moon illuminated for each seed‐removal and small‐mammal‐trapping session, because moon illumination can affect rodent antipredator behavior (e.g., Kotler et al., 1991; Orrock & Fletcher, 2014). We evaluated small‐mammal abundance and activity using Sherman live traps to survey the small‐mammal community at each study site during a three‐night session in 2014 (between 02 July and 10 July) and a five‐night session in 2015 (between 01 June and 14 June). We complemented small‐mammal trapping with camera trapping over 4 weeks including the 2015 seed‐removal session, using one motion‐activated trail camera at each site. See Appendix S1 for full details of these methods.

### 2.1 Quantifying seed removal by small mammals

To quantify seed removal, we deployed four seed depots per site during one 2‐week session in each of 2 years (21 July to 06 August 2014; 29 June to 15 July 2015). Seed depots consisted of translucent white plastic containers (21 × 13 cm; diameter × height) fitted with translucent lids in order to prevent predation by birds and large mammals and prevent loss of seeds from wind and rain (Mattos & Orrock, 2010; Mattos, Orrock, & Watling, 2013). Two 5‐cm^2^ openings cut on opposite sides allowed access by small mammals (Mattos et al., 2013). At each site, we placed one pair of depots, with less than 1 m between depots within a pair, in a location sheltered by vegetation, and a second pair, 1.5 m from the first, in an unsheltered location with relatively low vegetative cover to examine how small‐mammal use of vegetative cover might differ between areas with high and low expected predation risk due to differences in wolf occupancy. One depot in each pair allowed access by both small mammals and invertebrates (i.e., arthropods and mollusks), whereas the second depot in each pair allowed seed removal by invertebrates only by excluding small mammals via hardware cloth with 1.25 cm^2^ openings secured over each depot entrance (Appendix S1). By simultaneously measuring overall and invertebrate‐only seed removal, we were able to estimate the unique effect of small mammals. Ten seeds of each of four tree species were scattered on top of a layer of sand within each depot. To evaluate how removal differs among different seed species and sizes, we used seeds of *Acer saccharum* (54.77−98.95 mg; 95% CI), *Acer rubrum* (10.20−17.82 mg; 95% CI), *Tsuga canadensis* (2.88−3.44 mg; 95% CI) and *Betula alleghaniensis* (0.67−1.07 mg; 95% CI) purchased from commercial seed suppliers (Appendix S1). *Acer saccharum* and *A. rubrum* disperse seeds during or before the time during which we conducted seed‐removal trials, whereas *B. alleghaniensis* and *T. canadensis* disperse seeds in August—spring and August—September, respectively (i.e., starting 1 − 8 weeks after seed‐removal trials were conducted; Bonner & Karrfalt, 2008). All four of these tree species occurred within our study system, and basal area of these species did not differ significantly between high‐ and low‐wolf study sites (Table 1). For each seed‐removal session, depots were left in the field for 2 weeks (Bartowitz & Orrock, 2016) after which sand from each depot was sifted and intact seeds and seed fragments were counted (Mattos et al., 2013). Due to the novelty of the seed depot and/or the density of seeds in each depot to small mammals, the rates of seed removal observed may differ from background seed‐removal conditions. However, by placing seeds in a depot, we were able to standardize seed density and placement across study sites. This seed‐depot design has been used in previous studies to detect risk‐sensitive foraging differences between exposed and sheltered locations (Bartowitz & Orrock, 2016; Orrock et al., 2004; Orrock & Fletcher, 2014) and is therefore unlikely to affect our ability to detect differences in foraging due to predation risk. We use the term “seed removal” to describe seeds that were physically removed from depots (i.e., they were no longer present inside the depot) as well as seeds that were visibly destroyed within a tray (i.e., they were removed from the population of intact seeds). As small mammals can play a role in seed dispersal in addition to seed predation (Vander Wall, Kuhn, & Beck, 2005), we quantified the number of seeds consumed in situ (from empty seed coats and fragments left in depots) to compare the number of seeds removed to the number of seeds known to have been consumed (Appendix S4). This allowed us to evaluate the assumption, common in seed‐removal studies, that seed removal is generally indicative of seed predation (Mittelbach & Gross, 1984; Moles, Warton, & Westoby, 2003; Orrock et al., 2015).

**TABLE 1:** A comparison of small‐mammal community and habitat characteristics of high‐ and low‐wolf‐occupancy sites in Wisconsin (USA, 2014–2015)

Site characteristic	High‐wolf occupancy^a^	Low‐wolf occupancy^a^	*df*	*t*	*p*
Small‐mammal community					
Captures^b^, 2014	9.76 ± 2.06	24.04 ± 4.98	13.28	−2.65	.02
Captures^b^, 2015	2.70 ± 1.01	4.48 ± 2.06	14.47	−0.78	.45
Individuals^b^, 2014	8.51 ± 1.85	19.55 ± 4.18	13.74	−2.42	.03
Individuals^b^, 2015	1.98 ± 0.53	2.84 ± 1.31	13.18	−0.61	.55
Richness, 2014	2.10 ± 0.35	3.09 ± 0.31	19	−2.12	.05
Richness, 2015	1.30 ± 0.30	1.27 ± 0.33	19	0.06	.95
*P. leucopus* ^b^, 2014	3.50 ± 0.95	5.57 ± 1.76	19	−1.01	.33
*P. leucopus* ^b^, 2015	0.35 ± 0.18	0.32 ± 0.16	19	0.13	.90
*P. maniculatus* ^b^, 2014	1.77 ± 0.98	1.98 ± 0.89	19	−0.15	.88
*P. maniculatus* ^b^, 2015	0.35 ± 0.18	0.42 ± 0.18	19	−0.27	.79
*M. gapperi* ^b^, 2014	1.65 ± 0.61	8.78 ± 2.89	10.89	−2.41	.03
*M. gapperi* ^b^, 2015	0.69 ± 0.26	0.56 ± 0.39	19	0.28	.78
Habitat					
Canopy cover (%)	97.57 ± 0.71	98.03 ± 0.67	19	−0.48	.64
Total BA (cm^2^)	11,640.8 ± 875.2	14,452.1 ± 1,232.9	19	−1.83	.08
Shrub cover (%)	41.78 ± 7.70	51.71 ± 11.74	19	−0.69	.50
*A. saccharum* BA (cm^2^)	4,317.3 ± 1,171.3	6,120.6 ± 1649.7	19	−0.87	.39
*A. rubrum* BA (cm^2^)	1,198.4 ± 409.2	1,296.1 ± 528.2	19	−0.14	.89
*B. alleghaniensis* BA (cm^2^)	349.3 ± 229.0	287.1 ± 132.8	19	0.24	.81
*T. canadensis* BA (cm^2^)	525.7 ± 433.9	574.8 ± 574.8	19	−0.07	.95
Understory cover (%)	32.31 ± 5.13	37.12 ± 7.01	19	−0.54	.59
Litter depth (cm)	2.45 ± 0.19	2.45 ± 0.25	19	−0.07	.95
Canopy transmittance: 0.1 m (%)	5.05 ± 0.80	6.19 ± 1.40	19	−0.69	.50
Canopy transmittance: 1.5 m (%)	7.28 ± 2.49	7.05 ± 1.68	19	0.08	.94
Coarse woody debris (presence)	0.60 ± 0.16	0.64 ± 0.15	19	−0.16	.87

Note

We used wolf‐occupancy classifications from the same year in which each measurement was taken as wolf occupancy classification changed for two sites between the 2years of study.

^a^ Values for high‐ and low‐wolf occupancy are means ± *SE*.

^b^ Per 100 units of trap effort (Nelson & Clark, 1973).

### 2.2 Statistical analyses

Our hierarchical (split‐plot) design included wolf occupancy (high or low) applied at the site level, vegetative cover treatment (sheltered or unsheltered) or exclosure (small‐mammal access vs. small‐mammal exclosure) applied at the seed‐depot level, and seed species (*A. rubrum*, *A. saccharum*, *B. alleghaniensis*, or *T. canadensis*) at the within‐depot level. For analyses of seed removal, we used three linear mixed models (LMMs) with Gaussian response distributions (Littell, Milliken, Stroup, Wolfinger, & Schabenberger, 2006) after transforming the dependent variable (the proportion of seeds) using the logit transformation (Warton & Hui, 2011). Our analyses of the effect of wolf occupancy on overall seed removal and in situ seed consumption in 2014 and 2015 used LMMs with year, wolf occupancy, vegetative cover treatment, seed species, and all possible interactions as fixed effects. For analysis of seed removal in small‐mammal access versus small‐mammal exclosure depots, we pooled seed removal across cover treatments to simplify the model and included year, wolf occupancy, exclosure treatment, seed species, and all possible interactions as fixed effects. Our design includes multiple sources of variation that are important to accommodate into our analyses (Hurlbert, 1984). As such, we modeled site as a random effect and estimated three separate sources of site‐level variation, corresponding to the different levels of replication in our design: We estimate variation of sites within wolf occupancy areas, variation of sites among wolf occupancy areas among years, and variation of sites among wolf occupancy areas among years and vegetative cover or exclosure levels (Littell et al., 2006; Pinheiro & Bates, 2000).

We used *t* tests to compare average percent moon illumination between seed‐removal and small‐mammal‐trapping sessions as well as site‐level habitat characteristics between high‐ and low‐wolf‐occupancy sites including measures of vegetation characteristics, refuge availability, small‐mammal captures and unique small mammals captured per 100 units of trap effort (Nelson & Clark, 1973; Slade & Blair, 2000; Appendix S1), small‐mammal species richness, and proportion of small mammals recaptured. If unequal variance was detected, Satterthwaite's approximation was used (Littell, Stroup, & Freund, 2002). The number of unique small‐mammal individuals captured (M_t+1_) is highly correlated with abundance estimates from mark–recapture models (Slade & Blair, 2000), though due to our low‐recapture rates, especially in 2015, we chose to analyze only differences in the number of unique small mammals captured. We used generalized linear mixed models (GLMMs) with a binomial distribution and random effect for site to examine the correlations between the proportion of seeds removed and small‐mammal abundance.

All analyses were performed with SAS version 9.4 (Littell et al., 2006). Observations from 27 seed depots (including one entire site) were excluded from analyses due to disturbance (likely by black bears, *Ursus americanus*) or failure of small‐mammal exclosure (two observations in 2014; see Appendix S1 for details of excluded observations). For all analyses, we evaluated residual plots (residuals vs. linear predictor, histogram and box plot of residuals, and Q‐Q plot) to ensure that model assumptions were not violated. The full results of LMMs and contrasts are reported in Table 2 and Appendix S2, Table A1.

**TABLE 2:** Analysis of variance table of the results of linear mixed models using data from 2014 and 2015 seed‐removal depots for the proportion of seeds removed or destroyed in situ by small mammals and invertebrates together

Effect	ndf, ddf	*F*‐ratio	*p*
Wolf occupancy	1, 17	10.38	<.01
Year	1, 17	3.15	.09
Wolf occupancy × year	1, 17	0.17	.68
Cover	1, 32	1.98	.17
Cover × wolf occupancy	1, 32	0.66	.42
Cover × year	1, 32	2.02	.16
Cover × wolf occupancy × year	1, 32	0.06	.81
Species	3, 207	32.84	<.01
Species × wolf occupancy	3, 207	3.00	.03
High versus low wolf, *A. saccharum*	1, 207	12.26	<.01
High versus low wolf, *A. rubrum*	1, 207	12.50	<.01
High versus low wolf, *T. canadensis*	1, 207	7.96	<.01
High versus low wolf, *B. alleghaniensis*	1, 207	2.35	.13
Among species, high wolf	3, 207	7.49	<.01
Among species, low wolf	3, 207	30.97	<.01
Species × year	3, 207	2.59	.05
2014 versus 2015, *A. saccharum*	1, 207	5.88	.02
2014 versus 2015, *A. rubrum*	1, 207	4.41	.04
2014 versus 2015, *T. canadensis*	1, 207	1.45	.23
2014 versus 2015, *B. alleghaniensis*	1, 207	0.42	.52
Among species, 2014	3, 207	23.72	<.01
Among species, 2015	3, 207	10.81	<.01
Species × wolf occupancy × year	3, 207	0.24	.87
Species × cover	3, 207	0.31	.82
Species × wolf occupancy × cover	3, 207	0.06	.98
Species × year × cover	3, 207	0.91	.44
Species × wolf occupancy × year × cover	3, 207	0.30	.83

Note

Linear contrasts were used to examine significant interactions.

## 3 RESULTS

**FIGURE 2:**
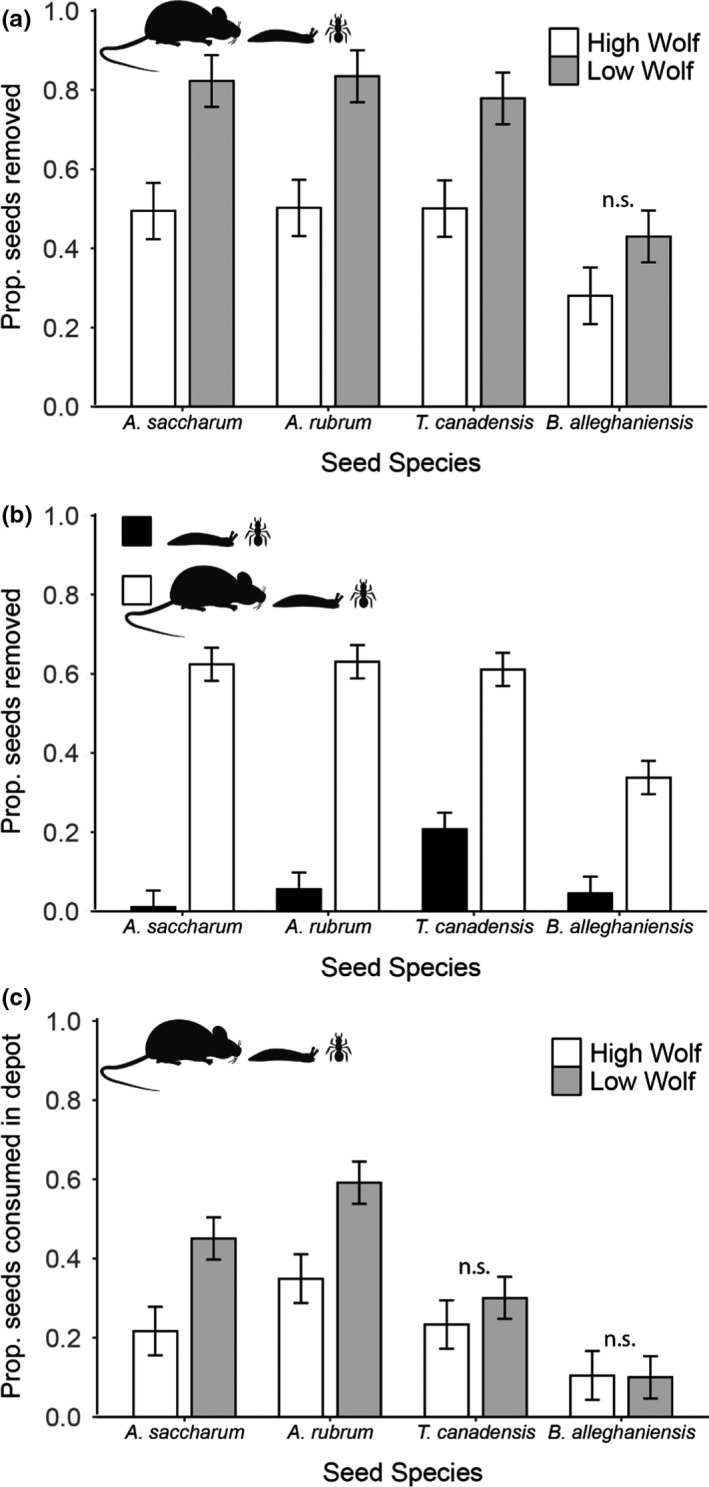
Mean proportions of *Acer saccharum*, *A. rubrum*, *Tsuga canadensis*, and *Betula alleghaniensis* seeds (a) removed or destroyed in situ by small mammals and invertebrates in high‐ and low‐wolf‐occupancy areas between June and August of 2014 and 2015, (b) removed or destroyed in situ by small mammals and invertebrates together (open bars) and invertebrates only (i.e., from depots excluding small mammals; closed bars) in 2014 and 2015, and (c) observed to be consumed in situ (within seed depots) in high‐ and low‐wolf‐occupancy areas during 2014 and 2015. Error bars represent one standard error above and below the mean. Logit‐transformed values were used in the analysis of seed removal, and nontransformed values are presented. The absence of a significant difference (*p* > .05) between two means is indicated by “n.s.” above a pair of bars

Over two 2‐week seed‐removal sessions, 58.70% (± 2.28% *SE*) of all seeds were removed from seed depots allowing access by both small mammals and arthropods. Of those seeds that were removed from all depots, most (86.44 ± 5.50% *SE*) were removed from depots allowing access by small mammals (Figure 2b). Game cameras aimed at seed depots during the 2015 removal session detected seed removal by small‐bodied mammals such as *Peromyscus* spp. and *Myodes gapperi* but did not detect any seed removal by larger mammals (e.g., squirrels) or birds (unpublished data). Destroyed and consumed seeds were observed in 81.33% (± 3.93% *SE*) of seed depots from which seeds had been removed and allowed access by small mammals (Appendix S4). There was a strong, positive relationship between seed removal and the number of seeds that were destroyed (*r* ^2^ > .4 and *p* < .01 for all species; Appendix S4), and estimates of in situ seed destruction were high: On average, 43.09 ± 2.19% *SE* of the seeds that were classified as removed were consumed (i.e., destroyed) within a tray (Figure 2c; Appendix S4).

Seed removal varied with wolf occupancy, seed species, and the interaction of wolf occupancy and seed species (Table 2; Figure 2a). A linear contrast of the interaction of wolf occupancy and seed species indicated that fewer seeds of *A. saccharum* (32.82 ± 9.69% difference), *A. rubrum* (33.21 ± 9.69% difference), and *T. canadensis* (27.82 ± 9.69% difference), but not *B. alleghaniensis* (14.95 ± 9.69% difference), were removed at high‐wolf sites (Table 2, Figure 2a). Seed removal differed marginally between 2014 and 2015 and the interaction between seed species and year was marginally significant, with a larger proportion of seeds per depot removed in 2014 for the two *Acer* species (Table 2, Appendix S2, Figure A2). Analysis of the difference between seed removal from depots with different exclosure treatments indicates a highly significant difference in seed removal between depots that allowed and excluded small mammals (*F*_1,34_ = 98.19, *p* < .01; Figure 2b) when seed removal was averaged across cover treatments (Appendix S2, Table A1a).

Results of our analysis of seeds consumed in situ indicated that wolf occupancy influences the number of *A. saccharum* and *A. rubrum* seeds consumed in depots for these larger‐seeded species: Greater numbers of *Acer* seeds were consumed in low‐wolf areas compared to areas with high‐wolf occupancy (Figure 2c). The proportion of seeds consumed in situ was significantly influenced by seed species (*F*_1,213_ = 40.30, *p* < .01); the interaction of species and wolf occupancy (*F*_1,213_ = 5.79, *p* < .01); the interaction of species and year (*F*_1,213_ = 6.74, *p* < .01); and the interaction of species, wolf occupancy, and year (*F*_1,213_ = 3.02, *p* = .03; Figure 2c). Linear contrasts of the interaction between species and wolf occupancy indicate that there was a significant difference in seeds consumed in situ between areas with high‐ and low‐wolf occupancy for the two *Acer* species (*A. saccharum*: *F*_1,213_ = 7.25, *p* < .01, difference = 23.40 ± 8.14%; *A. rubrum*: *F*_1, 213_ = 8.58, *p* < .01, difference = 24.20 ± 8.14%), but not *T. canadensis* (*F*_1, 213_ = 0.71, *p* = .40) or *B. alleghaniensis* (*F*_1, 213_ = 0.02, *p* = .90). Linear contrasts of the interaction between species, wolf occupancy, and year indicate that there was a significant difference in seeds consumed in situ between high‐ and low‐wolf occupancy for the two *Acer* species in 2014 (*A. saccharum*: *F*_1, 213_ = 11.71, *p* < .01; *A. rubrum*: (*F*_1,213_ = 14.52, *p* < .01), but not for in situ consumption in 2014 or any species in 2015 (all linear contrasts *p* > .05; Appendix S2, Table A1b).

The most common species captured during small‐mammal surveys were the southern red‐backed vole, *Myodes gapperi*, white‐footed mouse, *Peromyscus leucopus*, and deer mouse, *P. maniculatus*. The number of unique small‐mammal individuals captured per site differed annually (*t*_23.01_ = 4.38, *p* < .01), with an average of 14.29 ± 2.61 *SE* individuals captured per a site in 2014 and an average of 2.43 ± 0.72 *SE* individuals captured per site in 2015. During 2014, small mammals were more abundant in low‐wolf sites: The number of small‐mammal individuals, the total number of small‐mammal captures, and species richness were all significantly higher in low‐wolf‐occupancy sites (Table 1). However, there was no difference in these three metrics between high‐ and low‐wolf‐occupancy sites in 2015 (Table 1) when total captures were significantly lower than in 2014 (*t*_25.41_ = 4.05, *p* < .01). When small‐mammal species were analyzed separately, abundance of both *P. leucopus* and *P. maniculatus* did not differ between high‐ and low‐wolf sites in 2014 and 2015 (Table 1; there was also no difference in *Peromyscus* spp. abundance pooled across species between high‐ and low‐wolf‐occupancy sites; 2014: *t*_19_ = −0.89, *p* = .38; 2015: *t*_19_ = −0.10, *p* = .92). However, *M. gapperi* abundance was greater in low‐wolf‐occupancy sites in 2014 compared to high‐wolf‐occupancy sites yet did not differ between high‐ and low‐wolf‐occupancy sites in 2015 (Table 1). The average proportion of small‐mammal individuals recaptured did not differ between high‐ and low‐wolf‐occupancy sites in 2014 for all small‐mammal species pooled (*t*_19_ = −0.93, *p* = .36), *M. gapperi* (*t*_12_ = −1.38, *p* = .19), or *Peromyscus* spp. (*t*_17_ = −1.18, *p* = .26; Figure A3). In 2015, due to overall low small‐mammal captures, we did not have sufficient recaptures to evaluate whether recapture differed between high‐ and low‐wolf‐occupancy areas (four recaptures in low‐wolf sites and zero recaptures in high‐wolf sites). Abundance of small mammals was positively correlated with removal of *A. saccharum* (*F*_1,19_ = 5.41, *p* = .03), *A. rubrum* (*F*_1,19_ = 4.42, *p* = .05), and *B. alleghaniensis* (*F*_1,19_ = 7.77, *p* = .01), but not *T. canadensis* (*F*_1,19_ = 2.68, *p* = .12) across both years of our study and averaged across cover treatments (Appendix S5).

## 4 DISCUSSION

Our results indicate that regional variation in patterns of seed removal by small mammals, across 2 years, is influenced by the spatial distribution of wolf territories. Our results suggest that, because seed removal and destruction were significantly different in areas of high‐ versus low‐wolf occupancy (Figure 2a,c; Appendix S4), knowledge of apex predator behavior and territory locations may help predict spatial variation in seed survival over a large geographic region. These patterns of granivory are consistent with predicted top‐down effects mediated by wolves (Figure 1a), as supported by differences in small‐mammal captures in 2014 and absence of differences in habitat characteristics (Table 1). Furthermore, because the effect of wolf occupancy on seed survival differed among seed species (Figure 2a), our results suggest that changes in small‐mammal seed removal in high‐ versus low‐wolf‐occupancy areas may influence patterns of tree community composition (Ostfeld et al., 1997; Schnurr et al., 2002, 2004). This variation in patterns of seed removal may be primarily mediated by changes in small‐mammal abundance (specifically *M. gapperi*), rather than risk‐mediated changes in small‐mammal behavior.

### 4.1 Apex predator territories delimit hotspots and coldspots of granivory

In finding that territory boundaries of apex predators can provide insight into multiyear regional variation in seed removal and in situ consumption by small mammals (Figure 2a,c; Appendix S4), our study demonstrates that knowledge of space use by apex predators may be important for understanding large‐scale variation in the strength of top‐down effects on important processes (e.g., granivory). An implication of our results is that territory boundaries of apex carnivores may provide an important lens through which to interpret spatial variation in the strength of trophic interactions. For example, if we had not explicitly considered the location of wolf territories in our study, we would have failed to detect hotspots and coldspots of granivory that are clearly present in the landscape, since our data would average across opposing effects of predators within and outside wolf territories. Indeed, the evidence for population‐level effects of mesocarnivore release in Wisconsin are limited when abundance data are aggregated by region or county (Crimmins & Van Deelen, 2019), but the influence of wolves on mesocarnivores is evident when examined at a more‐localized scale (Flagel et al., 2017). As such, our findings suggest that future studies that incorporate information regarding the spatial constraints on predator activity may help provide useful insight into other large‐scale patterns in cascade strength, such as differences in cascades among ecosystems (e.g., Halaj & Wise, 2001; Schmitz, Hambäck, & Beckerman, 2000; Shurin et al., 2002), across climates (Rodríguez‐Castañeda, 2013), or along latitudinal gradients (Dyer & Coley, 2002).

We did not find evidence that small‐mammal foraging behavior differed between high‐ versus low‐wolf areas: Seed removal within a site was not greater in sheltered microhabitats, and the effect of wolf occupancy on seed removal did not differ between sheltered and unsheltered microhabitats (Table 2). This lack of response to cover may be because naturally occurring cover was widely available at all our study sites (e.g., vegetative cover and coarse woody debris; Table 1), even at relatively short distances from low‐cover depots. Furthermore, recapture probability, which might indicate changes in small‐mammal behavior, did not vary with wolf occupancy. However, more intensive small‐mammal surveys over multiple seasons may provide additional insight into the effects of predator interactions on small‐mammal community dynamics and contribute to understanding of contexts where the influences of wolf occupancy on granivory and seed survival are strongest.

We found no differences among habitat characteristics between high‐ and low‐wolf‐occupancy sites that could suggest alternative mechanisms to explain the observed patterns of granivory (Table 1). For example, vegetative cover differences between high‐ and low‐wolf areas could influence the predation risk perceived by small mammals (Flowerdew & Ellwood, 2001); however, we detected no differences in vegetative cover or light penetration between high‐ and low‐wolf‐occupancy sites that would support this alternative mechanism. Another possible alternative mechanism is that felid or mustelid carnivores may have affected small‐mammal abundance or activity. However, habitat characteristics that can predict the distribution of felids and mustelids (Gilbert, Wright, Lauten, & Probst, 1997; Lovallo & Anderson, 1996) did not differ between areas with high‐ and low‐wolf occupancy (Table 1). Although we did not estimate abundance or activity of coyotes and foxes directly at our exact study location, Flagel et al. (2017) found coyote activity decreases and fox activity increases in high‐wolf‐use areas at a study location within our study system. This significant link between wolves and the activity of coyotes and foxes, when considered in light of the absence of significant differences in an array of habitat characteristics between high‐ and low‐wolf sites, further suggests that increased seed survival in high‐wolf‐occupancy areas results from interactions among wolves, coyotes, and foxes.

### 4.2 Spatial variation in seed removal may yield spatial variation in tree communities

Our study indicates that the reduced granivory by small mammals in areas with high‐wolf occupancy may lead to differences in tree seedling recruitment and plant community composition inside wolf territories. Since small mammals are the primary seed predators in our study system (Crawley, 2000; Hulme, 1998; Whelan et al., 1991), capable of influencing plant community dynamics (Bricker & Maron, 2012; Brown & Heske, 1990; Howe & Brown, 2000; Maron et al., 2012; Orrock, Danielson, Burns, & Levey, 2003), variation in small‐mammal granivory based upon apex predator distribution can contribute to increased rates of change in plant community composition (Gordon et al., 2017). While secondary seed dispersal by granivores can be important in some systems and for some plant species (Vander Wall et al., 2005), our observations of seed fragments and consumed seeds in the majority of seed depots that allowed access by small mammals (81.33%; Appendix S4), the strong relationship between seed removal and destruction (Appendix S4), and results of other studies of seed fate (Abbott & Quink, 1970; Hsia & Francl, 2009) suggest that seed removal captures significant variation in seed predation for the species we studied.

We observed lower granivory in high‐wolf‐occupancy areas for seeds of the three largest‐seeded species in our study, but wolf occupancy did not significantly influence the removal of *B. alleghaniensis,* the smallest seed species tested, which was removed at lower rates than the other species. The lack of preference for *B. alleghaniensis* by small‐mammal granivores may negate differences in *B. alleghaniensis* consumption between high‐ and low‐wolf habitats (Figure 2a). Consequently, recruitment of tree species that produce seeds preferred by small‐mammal granivores (Abbott, 1962; Drickamer, 1970; Lobo, Duong, & Millar, 2009; Moles et al., 2003) may be higher in areas with high‐wolf occupancy (Brown & Heske, 1990; Maron et al., 2012; Whelan et al., 1991). Understanding regional‐scale variation in granivory as a result of apex predator distribution may enable prediction of where predation of large‐seeded species is greatest.

Our finding that wolf occupancy explains patterns of small‐mammal granivory complements and expands upon other studies that have documented differences in plant communities that arise from effects of wolf occupancy on ungulate herbivory (e.g., Callan, 2010; Callan, Nibbelink, Rooney, Wiedenhoeft, & Wydeven, 2013; Flagel, Belovsky, & Beyer, 2016). Our study suggests effects of wolf occupancy on seed predation by small mammals may help explain differences in plant community composition between high‐ and low‐wolf areas formerly attributed exclusively to the effects of deer herbivory. For example, *A. saccharum* was observed at higher seedling densities in high‐wolf areas even though it is relatively resistant to deer browse (Callan, 2010). Increased *A. saccharum* seed survival in high‐wolf areas, mediated by reduced granivory (Figure 2), may have contributed to differences in *A. saccharum* seedling densities between high‐ and low‐wolf areas in this study (Callan, 2010). Therefore, the effects of wolf occupancy on plant community composition through ungulate herbivory (e.g., Callan et al., 2013; Flagel et al., 2016; Fortin et al., 2005) may be compounded by interactions among carnivores that influence granivory by small mammals, particularly for browse‐sensitive tree species that produce seeds preferred by small mammals (e.g., *A. rubrum* and *T. canadensis*). While we did not detect differences in vegetation structure or light availability between high‐ and low‐wolf sites (Table 1), our surveys were designed to quantify differences in vegetation structure that might influence perception of predation risk by small mammals. Intensive, multiscale vegetation surveys that have detected an increase in cover of shrubs and forbs in high‐wolf areas (Callan, 2010; Callan et al., 2013) may contrast with our results because we conducted our study in habitat dominated by *A. saccharum, A. rubrum,* and *A. balsamea*, whereas Callan et al. (2013) conducted their study at a larger scale (1,000 m^2^ per site) with greater replication (32 sites) using the Carolina Vegetation Survey method (Peet, Wentworth, & White, 1998) in northern white cedar (*Thuja occidentalis*) wetlands, which are especially sensitive to deer herbivory (Habeck, 1960; Rooney, Solheim, & Waller, 2002; Van Deelen, Pregitzer, & Haufler, 1996). Further long‐term surveys of tree seedlings and other measures of plant recruitment coupled with seed‐removal studies may quantify variation in plant community composition due to differences in granivory between high and low‐wolf areas, especially as the duration of wolf occupancy of this region increases.

### 4.3 Changes in small‐mammal populations may drive changes in seed removal

Observations of lower seed removal and small‐mammal abundance in high‐wolf areas suggest that consumption of small mammals by mesopredators may help explain variation in patterns of small‐mammal granivory across wolf territories (Figure 1a). Overall, small‐mammal abundance and *M. gapperi* abundance were lower in high‐wolf sites in 2014 but not 2015 (Table 1). The interannual difference in the effect of wolf occupancy on small‐mammal abundance may be due to lower 2015 capture success that reflects annual fluctuations of small‐mammal populations with weather and resource availability (Ostfeld et al., 1996; Pucek et al., 1993; Wang et al., 2009), or temporal variation in small‐mammal behavior or abundance within a season, as 2015 small‐mammal surveys were conducted approximately 1 month earlier than 2014 surveys. While abiotic factors or bottom‐up effects (e.g., increased resource availability during an oak mast year; Ostfeld et al., 1996; Schnurr et al., 2002) can affect annual variation in small‐mammal abundance, we were able to detect differential seed predation between high‐ and low‐wolf sites in 2014 and 2015 despite much lower small‐mammal abundance and a corresponding drop in *Acer* spp. seed predation in 2015 compared to 2014, further demonstrating the utility of considering predator territoriality when predicting large‐scale patterns of seed predation. Reductions in small‐mammal captures and abundance, such as we observed in high‐wolf sites in 2014, correlate with increased survival of tree seeds in temperate forests (Ostfeld et al., 1997; Schnurr et al., 2002, 2004; Whelan et al., 1991). Indeed, across our study sites, seed removal was positively correlated with small‐mammal abundance even though we measured seed removal across a longer temporal scale than small‐mammal abundance (Appendix S5, Table A2). Annual variation in *Acer* spp. seed predation by small mammals may be related to differences in small‐mammal foraging activity due to increased moon illumination (Figure A2; Orrock et al., 2004; Wolfe & Summerlin, 1989); however, small‐mammal behavioral changes due to moon illumination cannot explain interannual differences in small‐mammal abundance as moon illumination did not differ between the 2014 and 2015 trapping sessions.

## 5 CONCLUSIONS

Our study provides evidence that large‐scale patterns of seed removal and consumption may exhibit significant variation between areas of high and low occupancy of apex predators: We found hotspots of small‐mammal activity and granivory in areas between wolf territories. Knowledge of apex predator territories can predict large‐scale, multiyear variation in granivory, which may affect plant community composition. Further studies of additional factors which may influence these interactions such as anthropogenic activity (Haswell, Kusak, & Hayward, 2017) and seasonal variation in food resources (Ostfeld et al., 1996; Schnurr et al., 2002) and/or abiotic conditions (Dhawan et al., 2018; Pucek et al., 1993; Wang et al., 2009) will be essential to furthering our understanding of the role that apex predators play in affecting small‐mammal granivory. For example, deep snow cover can inhibit the ability of red foxes to hunt small mammals (Halpin & Bissonette, 1988), which may disrupt the indirect effects of wolves on small‐mammal abundance, facilitating an increase in granivory by *M. gapperi* during winter. Hotspots and coldspots of seed predation delimited by territory boundaries may also predict patterns of ecological interactions other than granivory; for example, small mammals may be important hosts of zoonotic pathogens that affect humans (e.g., Levi, Kilpatrick, Mangel, & Wilmers, 2012). Studies of granivory may yield different results depending on whether spatial distribution of carnivores is considered, indicating that studies that consider effects of predators on ecosystem processes at the scale of carnivore territories may bring greater resolution to our understanding of spatial heterogeneity in biotic interactions.

## CONFLICT OF INTEREST

None declared.

## AUTHOR CONTRIBUTION

**Jennifer L. Chandler:** Conceptualization (equal); Data curation (lead); Formal analysis (lead); Funding acquisition (supporting); Investigation (lead); Methodology (equal); Writing‐original draft (lead); Writing‐review & editing (equal). **Timothy R. Van Deelen:** Conceptualization (equal); Project administration (supporting); Writing‐review & editing (supporting). **Nathan P. Nibbelink:** Conceptualization (supporting); Methodology (supporting); Writing‐review & editing (supporting). **John L. Orrock:** Conceptualization (equal); Formal analysis (supporting); Funding acquisition (lead); Methodology (equal); Project administration (lead); Writing‐original draft (supporting); Writing‐review & editing (equal).

## Supporting information

Appendix S1‐S5

## Data Availability

Data are available from the Dryad Digital Repository (https://doi.org/10.5061/dryad.stqjq2c12).
