# Supplementary material for: Large‐scale patterns of seed removal by small mammals differ between areas of low‐ versus high‐wolf occupancy
Source: Ecol Evol. 2020 Jun 1;10(14):7145–56. doi: 10.1002/ece3.6415 (PMC7391330; doi:10.1002/ece3.6415)
Supplement: Supplementary file 1 — Appendix S1‐S5 [file ECE3-10-7145-s001.docx]

# Supplementary Material

**Large-scale patterns of seed removal by small mammals differ between areas of low vs. high wolf occupancy**

Jennifer L. Chandler^1*^, Timothy R. Van Deelen^2^,

Nathan P. Nibbelink^3^ and John L. Orrock^1^

*1. Department of Integrative Biology, University of Wisconsin, Madison, WI 53706*

*2. Department of Forest and Wildlife Ecology, University of Wisconsin, Madison, WI 53706*

*3. Warnell School of Forestry and Natural Resources, University of Georgia, Athens, GA 30602*

^*^Corresponding author: *E-mail address:* [jchandler3@wisc.edu](mailto:chandlerjenniferl@gmail.com)

ORCID: <https://orcid.org/0000-0002-7266-520X>

# Appendix 1: Additional methods

## Wolf Occupancy Index

We assessed wolf occupancy using yearly presence or absence of wolves derived from minimum convex polygon estimates of resident wolf pack territories using radio locations of collared wolves and locations of sign and tracks (that excluded outlier points >5 km from other locations; Wisconsin Department of Natural Resources, Wydeven et al. 2009). We used three years of wolf-occupancy data to calculate the index since wolves can influence mesopredator aggregation within this length of time; wolves can reduce coyote density by 50% within three years after reintroduction (Crabtree and Sheldon 1999, Switalski 2003). Wolf occupancy classifications do not necessarily indicate wolf-occupancy per se at the time of our study but do indicate areas where wolf activity is expected to be higher or lower based upon territory delineations. Furthermore, our results for main effects of wolf occupancy on seed removal were not qualitatively different when five or ten years of wolf territory data was used. The following equation was used to calculate the duration-weighted wolf occupancy:

Duration-weighted wolf occupancy index $= Y_{t} + \frac{Y_{t-1}}{1.1} + \frac{Y_{t-2}}{1.2}$ (A1)

where “Y” is equal to 1 or 0 indicating the presence or absence of a wolf territory at a given location for year “t” (Callan 2010). To calculate the duration-weighted wolf-occupancy index, we summed three years of past wolf territory presence or absence (0 or 1 for each year), with more recent wolf territory presence weighted more heavily by dividing presence by a constant that increased by 0.1 with each year prior to the current year (sensu Callan 2010). This calculation resulted in an index ranging from 0 (no wolf occupancy over the past 3 years) to 2.74 (three years of past wolf occupancy), which has been successful in capturing meaningful variation in top-down effects in previous studies (Callan 2010). We chose sites equally distributed between locations with high-duration-weighted wolf occupancy (greater than 1.37, or 2 to 3 previous years of wolf occupancy) and low- duration-weighted wolf occupancy (less than 1.37, or 0 to 1 previous years of wolf occupancy), where there was a large natural break in the distribution of wolf occupancies by design (to maximize the difference between high and low wolf sites).

## Assessing habitat characteristics

To assess vegetation characteristics across high- and low-wolf occupancies during summer of 2014, we surveyed percent cover of understory vegetation (< 15 cm in height) and litter depth within eight 1.0 × 1.0 m quadrats. Shrub cover (woody vegetation > 15 cm in height, < 2.5 cm in stem diameter) was estimated within a 10 × 10 m area. Diameter at breast height (DBH) of trees greater than 2.5 cm DBH was measured within a 20 × 20 m area. We used a light sensor (Model LQM 70-10, Apogee Instruments, Inc.) to assess vegetative cover by measuring photosynthetically active radiation (PAR) between 0930 and 1500 hours between Jun and Aug of 2015. Three subsamples at both 0.1 m and 1.5 m above ground level and spaced 0.5 m apart, were taken at nine locations arranged on a 50 × 50 m, three by three grid across each site. Average percent light transmittance at each site was estimated by dividing average sub-canopy PAR by the average of ambient PAR measurements taken at the same height before and after sampling site PAR, in a location without shrub or canopy cover (Bolstad and Gower 1990). Most light measurements were taken on clear or partly cloudy days when clouds did not obstruct the sun (Ter-Mikaelian et al. 1999), but due to logistical constraints, light measurements at two sites were taken under overcast conditions. Presence or absence of coarse woody debris was estimated for woody debris larger than 2.5 cm in diameter within a 1.0 × 1.0 m area centered on each pair of seed depots. Differences in moon illumination between seed-removal and trapping sessions were examined using data tables of the fraction of the moon illuminated acquired from the Astronomical Applications Department of the United State Naval Observatory (http://aa.usno.navy.mil/data/docs/MoonFraction.php, accessed 06-Dec-2016) as moon illumination can influence patterns of woodland rodent activity (Mattos and Orrock 2010, Guiden and Orrock 2019).

## Small-mammal surveys

At each site, we deployed a three by three trapping grid with 25 m between trapping stations and two Sherman live traps (8 × 9 × 23 cm; H. B. Sherman Live Traps, Inc., Tallahassee, Florida) at each station. Traps were baited with whole oats, wrapped with insulation, and supplied with batting for nesting material. Each trapping session was preceded by one night of prebaiting to acclimate animals to the presence of traps. We checked traps once daily and replaced bait, batting, and insulation as needed. Upon capture, we marked each small mammal with a uniquely numbered ear tag and recorded the individual’s species, age, sex, reproductive status, and weight. We used ear length and weight measurements to distinguish between *P. leucopus* and *P. maniculatus* (Stephens et al. 2014).

Small mammals captured per 100 trap units was calculated using Nelson and Clark’s equation for correcting for sprung traps (due to disturbance and captured animals) in catch/effort calculations for trapping results:

$CE= \frac{A \times100}{{TU - IS}/2}$ (2)

where CE = catch per 100 trap units of effort, A = number of animals captured, TU = trap units (number of trap nights × number of traps), I = length of trapping interval, and S = total traps sprung (Nelson and Clark 1973). To calculate the number of small-mammal individuals captured per 100 trap units, we first calculated number of animals captured (M_t+1_; Slade and Blair 2000) and then adjusted M_t+1_ for sprung traps using the same equation but substituting M_t+1_ for number of animals captured (Nelson and Clark 1973).

## Quantifying seed removal by small mammals

Seed depots were secured with turf staples driven into the ground through the bottom of each bucket and filled with approximately 0.5 L of commercial sand (to the level of the bottom of the depot entrances). Seeds used in seed-removal assays were purchased from commercial seed suppliers (Sheffield’s Seeds, Locke, New York, USA; Treeseeds.com, Winooski, Vermont, USA; Lawyer Nursery, Inc, Plains, Montana, USA). These methods have been used previously to understand patterns of granivory (Mattos et al. 2013). Deployment and collection of seed depots were each spread over three days at the beginning and end of each seed removal session due to the distance between and logistics of traveling to each plot.

Our measure of seed removal includes seeds that were destroyed while in the depot as well as seeds that were removed from the depot and not recovered. As a result, seeds tallied as removed could experience one of three fates: known predation (i.e., seed destroyed in situ), removal and subsequent predation, or removal and subsequent survival. Because we did not track the fate of seeds removed from depots, we cannot distinguish which fraction of taken seeds survived (hence, our measure of removal includes seed mortality but also may indicate the potential for successful dispersal; see Appendix 4 for additional information on quantifying seed fate). Whereas overall seed removal by small mammals and invertebrates serves as an estimate of seed survival after primary dispersal to the forest floor and may inform our understanding of how differences in seed removal between high- and low-wolf areas influence plant recruitment. We used the logit transformation (Warton and Hui 2011) in our analyses of the proportion of seeds removed because we could not achieve convergence with binomial models of full complexity, however binomial models with reduced complexity yielded the same results as LMM models using logit-transformed data.

Although few depots were disturbed during the course of our study (25 of 176 depots), we excluded depots from analysis if they had been fully tipped over and/or moved >0.5 m from their original location. In cases where disturbance was slight (i.e., the depot was bumped but not moved and seeds were not spilled) we used observations from the depots if seed removal observed from the disturbed depot was within the 95% confidence interval of the mean of the non-disturbed depots within the same treatment group. All observations from one site were excluded from all analyses since depots were disturbed during both seed-removal sessions.

# Appendix 2: Results of linear mixed models

Table A1 ANOVA table for **a** the effect of wolf occupancy, year, seed species, and small mammal exclosure on the proportion of seeds removed from depots, pooled across cover treatments and **b** the effect of wolf occupancy, year, cover, and seed species on the proportion of seeds consumed in situ.

a)

| **Effect** | **ndf, ddf** | ***F*-ratio** | ***P*** |
| --- | --- | --- | --- |
| Wolf occupancy | 1, 14 | 12.22 | <0.01 |
| Year | 1, 14 | 2.93 | 0.11 |
| Wolf occupancy × year | 1, 14 | 1.03 | 0.33 |
| Exclosure | 1, 34 | 98.19 | <0.01 |
| Exclosure × wolf occupancy | 1, 34 | 10.68 | <0.01 |
| High vs. low wolf, small mammal access | 1, 34 | 22.90 | <0.01 |
| High vs. low wolf, small mammal exclosure | 1, 34 | 0.09 | 0.77 |
| Small mammal access vs. exclosure, high wolf | 1, 34 | 19.69 | <0.01 |
| Small mammal access vs. exclosure, low wolf | 1, 34 | 98.64 | <0.01 |
| Exclosure × year | 1, 34 | 1.38 | 0.25 |
| Exclosure × wolf occupancy × year | 1, 34 | 0.70 | 0.41 |
| Species | 3, 204 | 21.37 | <0.01 |
| Species × wolf occupancy | 3, 204 | 3.24 | 0.02 |
| High vs. low wolf, *A. saccharum* | 1, 204 | 12.48 | <0.01 |
| High vs. low wolf, *A. rubrum* | 1, 204 | 15.66 | <0.01 |
| High vs. low wolf, *T. canadensis* | 1, 204 | 8.30 | <0.01 |
| High vs. low wolf, *B. alleghaniensis* | 1, 204 | 1.93 | 0.17 |
| Among species, high wolf | 3, 204 | 6.05 | <0.01 |
| Among species, low wolf | 3, 204 | 20.26 | <0.01 |
| Species × year | 3, 204 | 2.80 | 0.04 |
| 2014 vs. 2015, *A. saccharum* | 1, 204 | 4.91 | 0.03 |
| 2014 vs. 2015, *A. rubrum* | 1, 204 | 2.86 | 0.09 |
| 2014 vs. 2015, *T. canadensis* | 1, 204 | 3.70 | 0.06 |
| 2014 vs. 2015, *B. alleghaniensis* | 1, 204 | 0.06 | 0.80 |
| Among species, 2014 | 3, 204 | 17.13 | <0.01 |
| Among species, 2015 | 3, 204 | 6.18 | <0.01 |
| Species × wolf occupancy × year | 3, 204 | 0.26 | 0.85 |
| Species × exclosure | 3, 204 | 16.94 | <0.01 |
| Small mammal access vs. exclosure, *A. saccharum* | 1, 204 | 121.00 | <0.01 |
| Small mammal access vs. exclosure, *A. rubrum* | 1, 204 | 96.07 | <0.01 |
| Small mammal access vs. exclosure, *T. canadensis* | 1, 204 | 45.91 | <0.01 |
| Small mammal access vs. exclosure, *B. alleghaniensis* | 1, 204 | 27.09 | <0.01 |
| Among species, Small mammal access | 3, 204 | 25.52 | <0.01 |
| Among species, Small mammal exclosure | 3, 204 | 12.79 | <0.01 |
| Species × wolf occupancy × exclosure | 3, 204 | 0.96 | 0.41 |
| Species × year × exclosure | 3, 204 | 3.66 | 0.01 |
| 2014 vs. 2015, *A. saccharum*, small mammal access | 1, 204 | 9.31 | <0.01 |
| 2014 vs. 2015, *A. saccharum*, small mammal exclosure | 1, 204 | 0.00 | 0.97 |
| 2014 vs. 2015, *A. rubrum*, small mammal access | 1, 204 | 5.39 | 0.02 |
| 2014 vs. 2015, *A. rubrum*, small mammal exclosure | 1, 204 | 0.00 | 0.97 |
| 2014 vs. 2015, *T. canadensis*, small mammal access | 1, 204 | 0.65 | 0.42 |
| 2014 vs. 2015, *T. canadensis*, small mammal exclosure | 1, 204 | 3.53 | 0.06 |
| 2014 vs. 2015, *B. alleghaniensis*, small mammal access | 1, 204 | 0.25 | 0.62 |
| 2014 vs. 2015, *B. alleghaniensis*, small mammal exclosure | 1, 204 | 0.71 | 0.40 |
| Among species, 2014, small mammal access | 3, 204 | 19.45 | <0.01 |
| Among species, 2014, small mammal exclosure | 3, 204 | 12.17 | <0.01 |
| Among species, 2015, small mammal access | 3, 204 | 8.66 | <0.01 |
| Among species, 2015, small mammal exclosure | 3, 204 | 2.92 | 0.03 |
| Small mammal access vs. exclosure, *A. saccharum*, 2014 | 1, 204 | 79.63 | <0.01 |
| Small mammal access vs. exclosure, *A. saccharum*, 2015 | 1, 204 | 42.93 | <0.01 |
| Small mammal access vs. exclosure, *A. rubrum*, 2014 | 1, 204 | 60.24 | <0.01 |
| Small mammal access vs. exclosure, *A. rubrum*, 2015 | 1, 204 | 36.53 | <0.01 |
| Small mammal access vs. exclosure, *T. canadensis*, 2014 | 1, 204 | 16.84 | <0.01 |
| Small mammal access vs. exclosure, *T. canadensis*, 2015 | 1, 204 | 30.71 | <0.01 |
| Small mammal access vs. exclosure, *B. alleghaniensis*, 2014 | 1, 204 | 17.48 | <0.01 |
| Small mammal access vs. exclosure, *B. alleghaniensis*, 2015 | 1, 204 | 9.89 | <0.01 |
| Species × wolf occupancy × year × exclosure | 3, 204 | 0.15 | 0.93 |

b)

| **Effect** | **ndf, ddf** | ***F*-ratio** | ***P*** |
| --- | --- | --- | --- |
| Wolf occupancy | 1, 19 | 3.60 | 0.07 |
| Year | 1, 19 | 1.21 | 0.28 |
| Wolf occupancy × year | 1, 19 | 2.84 | 0.11 |
| Cover | 1, 33 | 0.08 | 0.79 |
| Cover × wolf occupancy | 1, 33 | 0.12 | 0.74 |
| Cover × year | 1, 33 | 0.50 | 0.48 |
| Cover × wolf occupancy × year | 1, 33 | 0.05 | 0.83 |
| Species | 3, 213 | 40.30 | <0.01 |
| Species × wolf occupancy | 3, 213 | 5.79 | <0.01 |
| High vs. low wolf, *A. saccharum* | 1, 213 | 7.25 | <0.01 |
| High vs. low wolf, *A. rubrum* | 1, 213 | 8.58 | <0.01 |
| High vs. low wolf, *T. canadensis* | 1, 213 | 0.71 | 0.40 |
| High vs. low wolf, *B. alleghaniensis* | 1, 213 | 0.02 | 0.90 |
| Among species, high wolf | 3, 213 | 7.82 | <0.01 |
| Among species, low wolf | 3, 213 | 43.42 | <0.01 |
| Species × year | 3, 213 | 6.74 | <0.01 |
| 2014 vs. 2015, *A. saccharum* | 1, 213 | 1.73 | 0.19 |
| 2014 vs. 2015, *A. rubrum* | 1, 213 | 8,82 | <0.01 |
| 2014 vs. 2015, *T. canadensis* | 1, 213 | 0.62 | 0.43 |
| 2014 vs. 2015, *B. alleghaniensis* | 1, 213 | 0.05 | 0.83 |
| Among species, 2014 | 3, 213 | 34.95 | <0.01 |
| Among species, 2015 | 3, 213 | 11.41 | <0.01 |
| Species × wolf occupancy × year | 3, 213 | 3.02 | 0.03 |
| 2014 vs. 2015, *A. saccharum*, high wolf | 1, 213 | 0.31 | 0.58 |
| 2014 vs. 2015, *A. saccharum*, low wolf | 1, 213 | 7.06 | <0.01 |
| 2014 vs. 2015, *A. rubrum*, high wolf | 1, 213 | 0.10 | 0.75 |
| 2014 vs. 2015, *A. rubrum*, low wolf | 1, 213 | 17.36 | <0.01 |
| 2014 vs. 2015, *T. canadensis*, high wolf | 1, 213 | 0.44 | 0.51 |
| 2014 vs. 2015, *T. canadensis*, low wolf | 1, 213 | 0.19 | 0.66 |
| 2014 vs. 2015, *B. alleghaniensis,* high wolf | 1, 213 | 0.16 | 0.69 |
| 2014 vs. 2015, *B. alleghaniensis*, low wolf | 1, 213 | 0.63 | 0.43 |
| Among species, 2014, high wolf | 3, 213 | 5.10 | <0.01 |
| Among species, 2014, low wolf | 3, 213 | 45.27 | <0.01 |
| Among species, 2015, high wolf | 3, 213 | 3.10 | 0.03 |
| Among species, 2015, low wolf | 3, 213 | 9.62 | <0.01 |
| High vs. low wolf, *A. saccharum*, 2014 | 1, 213 | 11.71 | <0.01 |
| High vs. low wolf, *A. saccharum*, 2015 | 1, 213 | 0.27 | 0.60 |
| High vs. low wolf, *A. rubrum*, 2014 | 1, 213 | 14.52 | <0.01 |
| High vs. low wolf, *A. rubrum*, 2015 | 1, 213 | 0.23 | 0.64 |
| High vs. low wolf, *T. canadensis*, 2014 | 1, 213 | 0.58 | 0.45 |
| High vs. low wolf, *T. canadensis*, 2015 | 1, 213 | 0.23 | 0.63 |
| High vs. low wolf, *B. alleghaniensis*, 2014 | 1, 213 | 0.42 | 0.52 |
| High vs. low wolf, *B. alleghaniensis*, 2015 | 1, 213 | 0.22 | 0.64 |
| Species × cover | 3, 213 | 0.40 | 0.75 |
| Species × wolf occupancy × cover | 3, 213 | 0.10 | 0.96 |
| Species × year × cover | 3, 213 | 0.41 | 0.75 |
| Species × wolf occupancy × year × cover | 3, 213 | 0.33 | 0.80 |

**
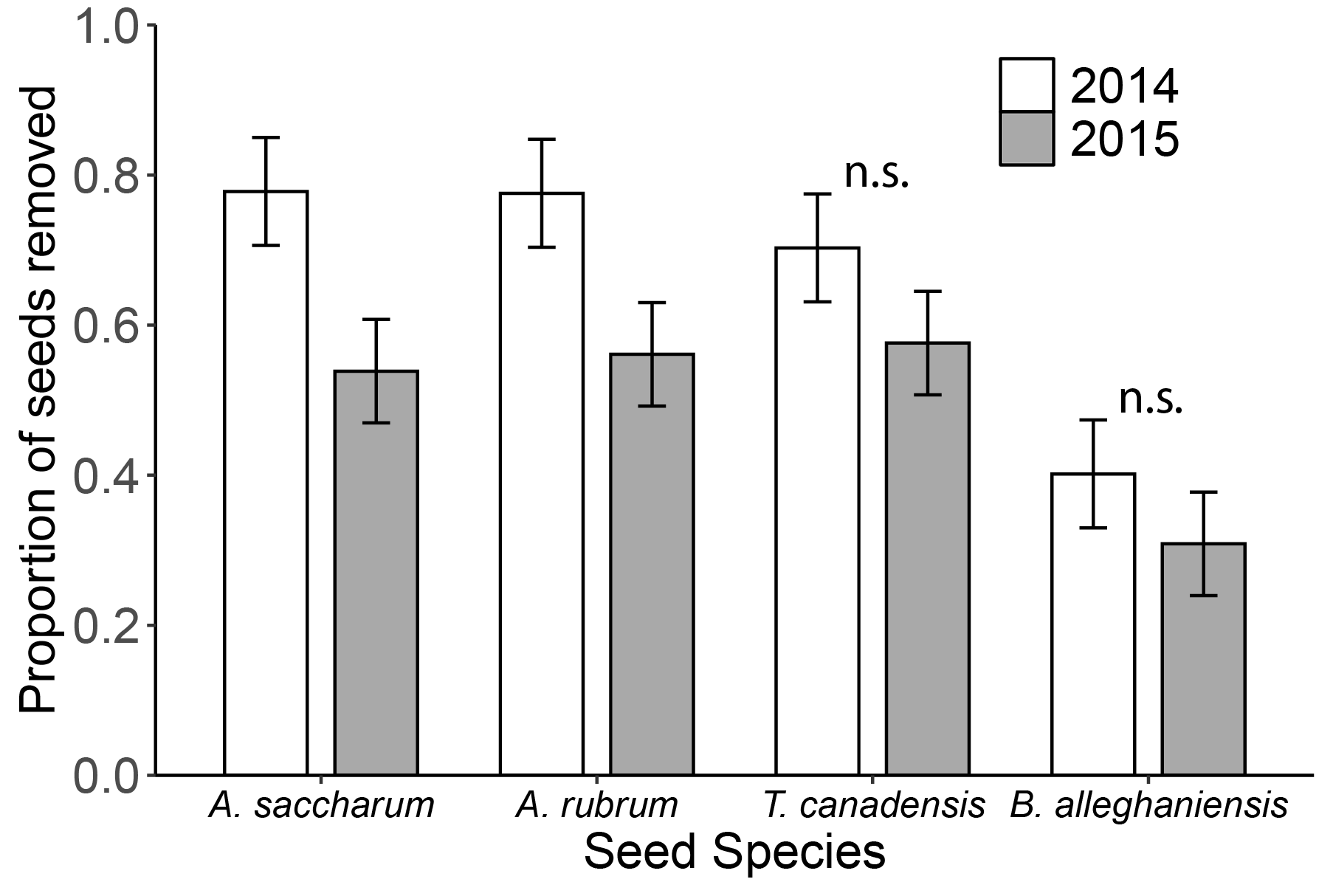
**

**Fig. A2** Mean proportion of *Acer saccharum*, *A. rubrum*, *Tsuga canadensis*, and *Betula alleghaniensis* seeds removed from depots allowing access by small mammals and invertebrates during Jun−Aug of 2014 and 2015. Error bars represent one standard error above and below the mean. The absence of a significant difference (*P* > 0.05) between high- and low-wolf sites is indicated by “n.s.” above a pair of bars.

# Appendix 3. Small-mammal recapture probability

**
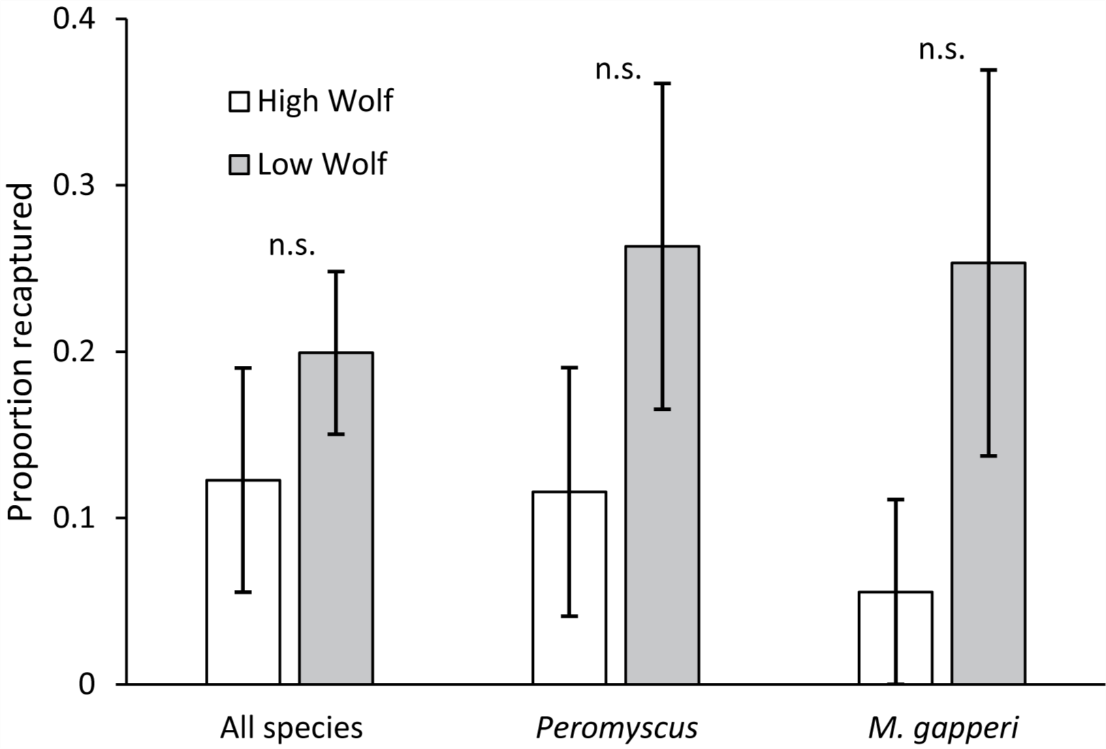
**

**Fig. A3** Average proportion of small mammals (all species pooled, *Peromyscus* spp., and *M. gapperi*) recaptured in 2014. Sites without any small-mammal captures in 2014 are excluded. Error bars represent one standard error above and below the mean. The absence of a significant difference (*P* > 0.05) between high- and low-wolf sites is indicated by “n.s.” above a pair of bars.

# Appendix 4: Quantifying known seed predation rates

In order to examine the proportion of seeds removed from depots that had potential to be cached by small mammals and later germinate (i.e., the number of seeds with an unknown fate; Vander Wall et al. 2005), we counted seed fragments and empty seed coats left in each depot at the end of each seed removal session. A single seed fragment was only included in our count if it was equal to more than 50% of an entire seed in order to avoid counting a single consumed seed more than once. In particular, *Acer* spp. seeds consumed by small mammals were easily identified as such due to the characteristic way in which small mammals destroy one edge of the seed coat and remove the seed inside. This method of seed consumption leaves the samara and much of the seed coat intact, making it possible to quantify the number of destroyed seeds with a high degree of confidence (Fig. A4). We observed evidence of seeds consumed in situ in 81.33 ± 3.93% of seed depots that allowed access by small mammals and arthropods from which seeds had been removed (i.e., excluding depots from which no seeds were removed); we observed evidence of *A. saccharum* consumed in 82.81 ± 4.75% SE, *A. rubrum* in 89.06 ± 3.93% SE, *T. canadensis* in 71.01 ± 5.50% SE, and *B. alleghaniensis* in 48.44 ± 6.30% SE of small mammal and arthropod depots, considering only depots from which a given species was removed (Fig. A5). Of all seeds tallied as removed from depots allowing small mammals, 43.09 ± 2.19% SE (49.18 ± 4.52% SE of *A. saccharum* seeds, 66.64 ± 4.00% SE of *A. rubrum* seeds, 37.05 ± 3.75% SE of *T. canadensis* seeds, and 19.95 ± 3.05% SE of *B. alleghaniensis*) were recovered as empty seed coats or fragments of seeds where the seed embryo had been damaged, indicating that they had been consumed in situ (Fig. A6). The number of seeds tallied as removed from a depot (i.e., handled by a granivore) and the number of seeds observed to be destroyed within a depot were strongly positively correlated (*A. saccharum*: r^2^ = 0.45, d.f. = 78, *P* < 0.01; *A. rubrum*: r^2^ = 0.70, d.f. = 78, *P* < 0.01; *T. canadensis:* r^2^ = 0.41, d.f. = 78, *P* < 0.01; *B. alleghaniensis*: r^2^ = 0.53, d.f. = 78, *P* < 0.01 ). Our count of remains of seeds consumed in depots is a minimum count of the number of seeds destroyed by granivores, as we only searched for and counted seeds within depots, which does not account for seeds consumed immediately outside depots, seeds taken to other refuges for consumption, or seeds cached and later consumed. Additional studies suggest that the fate of many removed seeds is likely to be death, as 100% of the seeds cached by *Myodes gapperi* and *Peromyscus leucopus* in a study in western Massachusetts were consumed within three weeks (Abbott and Quink 1970). The pattern of increased seed removal in areas with low-wolf occupancy was likely driven by the differences in in situ seed consumption across wolf territories, as the influence of wolf occupancy on seeds removed from seed depots intact (i.e., seeds with unknown fate) is not significant (*F*_1, 19_ = 0.13, *P* = 0.72).

Our observations of seeds consumed in situ indicate that more than half of *Acer* seeds (the species for which consumption was most reliably estimated; Fig. A4) handled by small mammals are consumed immediately in seed depots (Supplementary material Appendix 4; Fig. A6). Of the remaining half of seeds handled by small mammals with an unknown fate, few seeds were likely to be successfully dispersed; within our study system, Hsia and Francl (2009) observed that only 5% of *A. saccharum* seeds recovered after removal by small mammals were cached during the summer months. Of those 5% of seeds that were potentially cached, it is unlikely that any survived to germination, as Abbot and Quink (1970) found that 100% of caches made by *P. leucopus* and *M. gapperi* during the summer months were revisited and completely destroyed within three weeks. Furthermore, while scatter-hoarding behavior of *Peromyscus* spp. has been well documented (e.g., Barry 1996; Vander Wall et al. 2001; Plucinski and Hunter 2001), our small-mammal trapping results suggest that differences in seed removal by small mammals between high- and low-wolf areas are due to the changes in *M. gapperi* abundance, not *Peromyscus* spp. Evidence suggests that *M. gapperi* either immediately consumes seeds (Schnurr et al. 2002) or caches a much lower percentage of seeds than *Peromyscus* spp. (Plucinski and Hunter 2001). While our results demonstrate that both seed removal and in situ consumption of seeds differed with wolf occupancy (Fig. 2a, 2c; Table A1b), our results cannot inform the ultimate fate of intact seeds that were removed (i.e., these may represent dispersal events or eventual mortality). Importantly, when we examine only observations of seeds that were destroyed in situ (i.e., known mortality events), the pattern of reduced seed predation of *Acer* spp. seeds in high-wolf areas remains significant (Table A1b). As a result, whether we consider the role of small mammals as seed predators or both seed predators and dispersers, our findings demonstrate that these important small mammal activities are influenced by wolf occupancy.


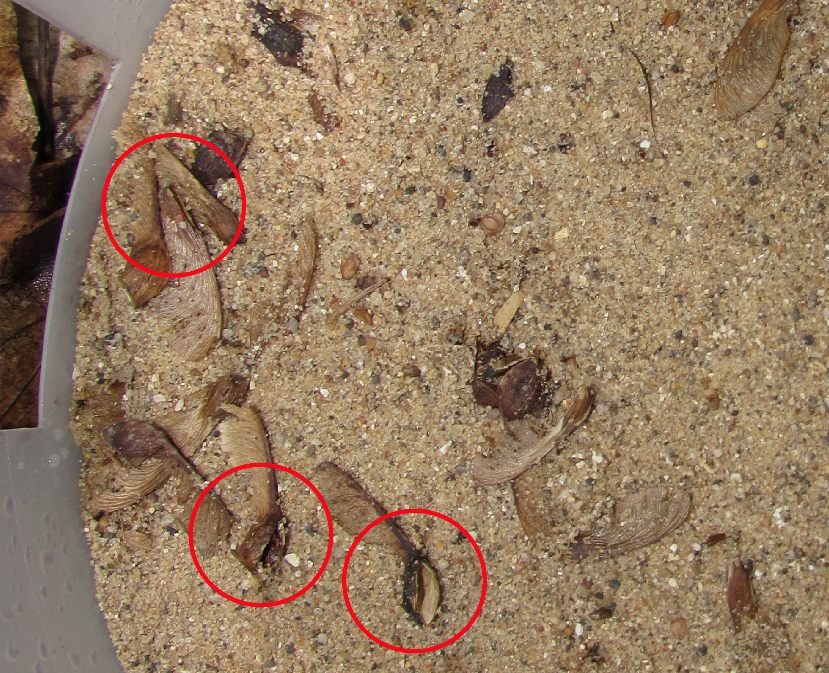


**Fig. A4** Photograph of *Acer saccharum* and *A. rubrum* seeds consumed by small mammals inside a seed depot (i.e., in situ consumption) during the 2014 seed removal session. Red circles indicate examples of consumed seeds.


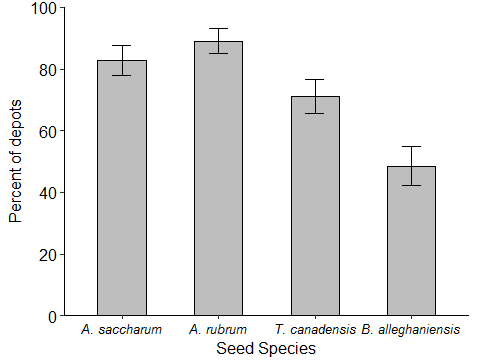


**Fig. A5** Average percent of depots allowing access by small mammals and arthropods with evidence of seed consumption. Error bars represent one standard error above and below the mean.


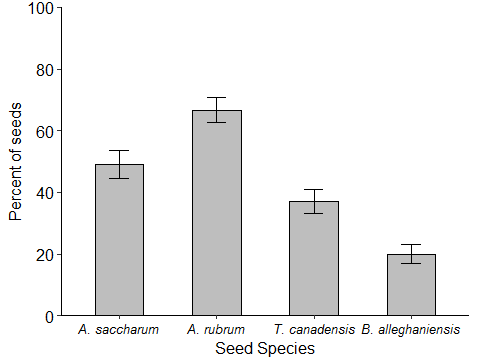


**Fig. A6** Mean percent of seeds tallied as “removed” that were observed to be consumed in depots allowing access by small mammals and arthropods. Error bars represent one standard error above and below the mean.

# Appendix 5: Correlations between seed removal and small mammal abundance

Table A2: Results of generalized linear mixed models (GLMMs) examining the influence of *Myodes gapperi* and *Peromyscus* spp. abundance (M_t+1_) on the proportion of seeds removed during both years of study. Models used a binomial distribution and random effect for site. The response variables, proportion of seeds removed for a given species, are averaged across cover treatment.

| **Response Variable** |  | **Effect** | **ndf, ddf** | ***F*-ratio** | ***P*** |
| --- | --- | --- | --- | --- | --- |
| Seed removal of all 4 species |  | Small mammal M_t+1_ | 1, 19 | 21.63 | <0.01 |
| *A. saccharum* seed removal |  | *M. gapperi* M_t+1_ | 1, 19 | 4.40 | 0.05 |
| *A. rubrum* seed removal |  | *M. gapperi* M_t+1_ | 1, 19 | 4.02 | 0.06 |
| *T. canadensis* seed removal |  | *M. gapperi* M_t+1_ | 1, 19 | 1.91 | 0.18 |
| *B. alleghaniensis* seed removal |  | *M. gapperi* M_t+1_ | 1, 19 | 4.28 | 0.05 |
| *A. saccharum* seed removal |  | *Peromyscus* M_t+1_ | 1, 19 | 5.72 | 0.03 |
| *A. rubrum* seed removal |  | *Peromyscus* M_t+1_ | 1, 19 | 4.44 | 0.05 |
| *T. canadensis* seed removal |  | *Peromyscus* M_t+1_ | 1, 19 | 4.14 | 0.06 |
| *B. alleghaniensis* seed removal |  | *Peromyscus* M_t+1_ | 1, 19 | 8.71 | <0.01 |

# Supplemental Literature Cited

Abbott, H. G., and T. F. Quink. 1970. Ecology of eastern white pine seed caches made by small forest mammals. Ecology 51:271–278.

Barry, W. J. 1996. Environmental effects on food hoarding in deermice (*Peromyscus*). Journal of Mammalogy 57:731–746.

Bolstad, P. V, and S. T. Gower. 1990. Estimation of leaf area index in fourteen southern Wisconsin forest stands using a portable radiometer. Tree Physiology 7:115–124.

Callan, R. 2010. Are wolves in Wisconsin affecting the biodiversity of understory plant communities via a trophic cascade? PhD Dissertation, The University of Georgia.

Callan, R., N. P. Nibbelink, T. P. Rooney, J. E. Wiedenhoeft, and A. P. Wydeven. 2013. Recolonizing wolves trigger a trophic cascade in Wisconsin (USA). Journal of Ecology 101:837–845.

Crabtree, R. L., and J. W. Sheldon. 1999. Coyotes and canid coexistence in Yellowstone. Pages 127–164 *in* T. W. Clark, A. P. Curlee, S. C. Minta, and P. M. Kareiva, editors. Carnivores in ecosystems: the Yellowstone experience. Yale University Press, New Haven.

Guiden, P. W., and J. L. Orrock. 2019. Invasive shrubs modify rodent activity timing, revealing a consistent behavioral rule governing diel activity. Behavioral Ecology 30:1069–1075.

Hsia, J. F., and K. E. Francl. 2009. Postdispersal sugar maple (*Acer saccharum*) seed predation by small mammals in a northern hardwood forest. The American Midland Naturalist 162:213–223.

Mattos, K. J., and J. L. Orrock. 2010. Behavioral consequences of plant invasion: an invasive plant alters rodent antipredator behavior. Behavioral Ecology 21:556–561.

Mattos, K. J., J. L. Orrock, and J. I. Watling. 2013. Rodent granivores generate context-specific seed removal in invaded and uninvaded habitats. The American Midland Naturalist 169:168–178.

Nelson, L., and F. W. Clark. 1973. Correction for sprung traps in catch/effort calculations of trapping results. Journal of Mammalogy 54:295–298.

Plucinski, K. E., and M. L. Hunter. 2001. Spatial and temporal patterns of seed predation on three tree species in an oak-pine forest. Ecography 24:309–317.

Schnurr, J. L., R. S. Ostfeld, and C. D. Canham. 2002. Direct and indirect effects of masting on rodent populations and tree seed survival. Oikos 96:402–410.

Slade, N. A., and S. M. Blair. 2000. An empirical test of using counts of individuals captured as indices of population size. Journal of Mammalogy 81:1035–1045.

Stephens, R. B., E. M. Anderson, S. R. Wendt, and J. K. Meece. 2014. Field identification of sympatric *Peromyscus leucopus noveboracensis* and *P. maniculatus gracilis* in Wisconsin from external measurements. The American Midland Naturalist 171:139–146.

Switalski, T. A. 2003. Coyote foraging ecology and vigilance in response to gray wolf reintroduction in Yellowstone National Park. Canadian Journal of Zoology 81:985–993.

Ter-Mikaelian, M. T., R. G. Wagner, F. W. Bell, and C. Shropshire. 1999. Comparison of photosynthetically active radiation and cover estimation for measuring the effects of interspecific competition on jack pine seedlings. Canadian Journal of Forest Research 29:883–889.

Vander Wall, S. B., K. M. Kuhn, and M. J. Beck. 2005. Seed removal, seed predation, and secondary dispersal. Ecology 86:801–806.

Vander Wall, S. B., T. C. Thayer, J. S. Hodge, M. J. Beck, and J. K. Roth. 2001. Scatter-hoarding behavior of deer mice (*Peromyscus maniculatus*). Western North American Naturalist 61:109–113.

Warton, D. I., and F. K. C. Hui. 2011. The arcsine is asinine: the analysis of proportions in ecology. Ecology 92:3–10.

Wydeven, A. P., J. E. Wiedenhoeft, R. N. Schultz, R. P. Thiel, R. L. Jurewicz, B. E. Kohn, and T. R. Van Deelen. 2009. History, population growth, and management of wolves in Wisconsin. Pages 87–105 *in* A. P. Wydeven, T. R. Van Deelen, and E. J. Heske, editors. Recovery of gray wolves in the Great Lakes region of the United States. Springer New York, New York, NY.
